# Aminoglycoside-Induced Cochleotoxicity: A Review

**DOI:** 10.3389/fncel.2017.00308

**Published:** 2017-10-09

**Authors:** Meiyan Jiang, Takatoshi Karasawa, Peter S. Steyger

**Affiliations:** ^1^Oregon Hearing Research Center, Oregon Health & Science University, Portland, OR, United States; ^2^National Center for Rehabilitative Auditory Research, Portland VA Medical Center (VHA), Portland, OR, United States

**Keywords:** aminoglycosides, gentamicin, ototoxicity, cochleotoxicity, nephrotoxicity, inflammation, systemic administration

## Abstract

Aminoglycoside antibiotics are used as prophylaxis, or urgent treatment, for many life-threatening bacterial infections, including tuberculosis, sepsis, respiratory infections in cystic fibrosis, complex urinary tract infections and endocarditis. Although aminoglycosides are clinically-essential antibiotics, the mechanisms underlying their selective toxicity to the kidney and inner ear continue to be unraveled despite more than 70 years of investigation. The following mechanisms each contribute to aminoglycoside-induced toxicity after systemic administration: (1) drug trafficking across endothelial and epithelial barrier layers; (2) sensory cell uptake of these drugs; and (3) disruption of intracellular physiological pathways. Specific factors can increase the risk of drug-induced toxicity, including sustained exposure to higher levels of ambient sound, and selected therapeutic agents such as loop diuretics and glycopeptides. Serious bacterial infections (requiring life-saving aminoglycoside treatment) induce systemic inflammatory responses that also potentiate the degree of ototoxicity and permanent hearing loss. We discuss prospective clinical strategies to protect auditory and vestibular function from aminoglycoside ototoxicity, including reduced cochlear or sensory cell uptake of aminoglycosides, and otoprotection by ameliorating intracellular cytotoxicity.

## Aminoglycoside Antibiotics

**Figure 1:**
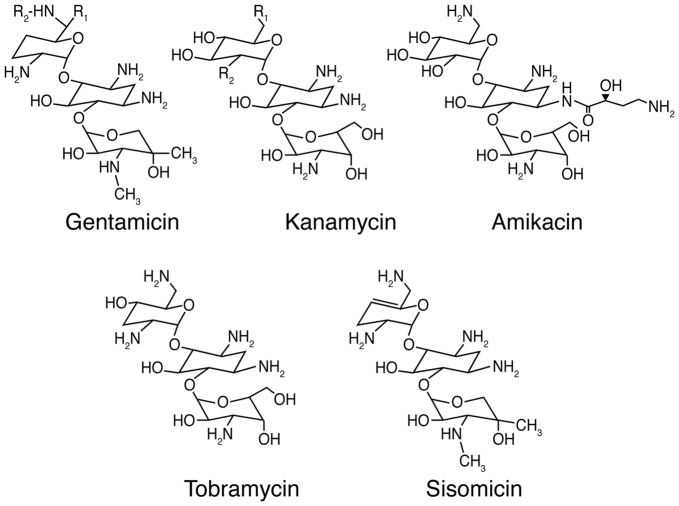
Chemical structures of selected aminoglycoside antibiotics. For gentamicin C_1_: R_1_ = R_2_ = CH_3_; gentamicin C_2_: R_1_ = CH_3_, R_2_ = H; and gentamicin C_1A_: R_1_ = R_2_ = H. For kanamycin A: R_1_ = NH_2_, R_2_ = OH; kanamycin B: R_1_ = R_2_ = NH_2_; and kanamycin C: R_1_ = OH, R_2_ = NH_2_.

Aminoglycosides are among the most efficacious antibiotics used to treat serious Gram-negative infections by *Pseudomonas*, *Salmonella* and *Enterobacter* species (Forge and Schacht, 2000). The first identified aminoglycoside, streptomycin, was isolated from *Streptomyces griseus* in 1944 (Schatz et al., 1944), followed by neomycin from *Streptomyces fradiae* (Waksman and Lechevalier, 1949). In 1957 and 1963, kanamycin and gentamicin (Figure 1) were isolated from *Streptomyces kanamyceticus* (Umezawa et al., 1957) and the actinomycete* Micromonospora purpurea* (Weinstein et al., 1963) respectively, followed by tobramycin from *Streptomyces tenebrarius* (Wick and Welles, 1967) and amikacin, a semi-synthetic derivative of kanamycin A (Kawaguchi et al., 1972). Aminoglycosides with the–mycin suffix are derived from *Streptomyces* genera, while those from *Micromonospora* genera have the suffix–micin. Aminoglycosides can also treat selected Gram-positive infections like tuberculosis due to the intracellular *Mycobacterium tuberculosis* (Forge and Schacht, 2000). Clinically, aminoglycosides are often used in combination with β-lactams (like ampicillin) for combinatorial synergistic efficacy against a broad range of bacteria, especially when the causative microbe(s) is unknown (Dressel et al., 1999), and has been well-characterized for *Pseudomonas* and other Gram-negative bacteria (Niederman et al., 2001).

Nonetheless, these drugs can induce acute dose-dependent kidney failure (nephrotoxicity), and permanent hearing loss (cochleotoxicity; defined here as hearing loss in the conventional frequency range, i.e., <8 kHz) and/or balance disorders (vestibulotoxicity). Aminoglycoside-induced vestibulotoxicity and/or cochleotoxicity occurs in as many as 20% of patients who received these drugs intravenously for multiple days (Ariano et al., 2008; Al-Malky et al., 2015; Garinis et al., 2017a). Hearing loss delays speech acquisition, education and psychosocial development, reducing employability, income and tax revenues (Jones and White, 1990; Järvelin et al., 1997; Mehl and Thomson, 1998; Naramura et al., 1999; Tambs, 2004), with a socioeconomic burden >$1,393,000 in 2015 dollars over the life-time of each pre-lingually deafened child (Mohr et al., 2000). Similarly, for each adult that acquires hearing loss, the socioeconomic burden is >$350,000 in 2015 dollars over their remaining lifespan.

The bactericidal efficacy of aminoglycosides against a broad range of bacteria is directly related to peak concentration in the blood. Yet aminoglycosides have a narrow therapeutic index, and it is crucial to maintain or enhance their therapeutic efficacy while minimizing their side effects. The increasing prevalence of bacterial resistance to more commonly-used antibiotics, e.g., ampicillin, β-lactams (Puopolo and Eichenwald, 2010; Tsai et al., 2014) has resulted in the retention of aminoglycosides as a clinically necessary alternative treatment. Aminoglycosides also remain an attractive clinical antibiotic strategy due to their chemical stability at ambient temperature (particularly in sub-Sahara Africa), rapid bactericidal effect, lower incidence of resistance, and relative lower cost compared to newer, synthetic, more costly non-ototoxic medications.

Advances in molecular biology have enabled the bactericidal mechanisms of aminoglycosides, and subsequent emergence of bacterial resistance to these drugs, to be studied extensively (Shakil et al., 2008). Furthermore, the emerging bioactivities and potential applications of aminoglycosides continue to be extensively investigated. For example, the K20 derivative of kanamycin A with an octanesulfonyl chain is a broad-spectrum antifungal that targets fungal plasma membranes to protect agricultural crops (Shrestha et al., 2014). Selected aminoglycosides are being tested for their ability to read-through premature stop-codons in genetic mutations for the cystic fibrosis transmembrane conductance regulator (CFTR) and selected cancers (Du et al., 2006; Baradaran-Heravi et al., 2017).

Currently nine aminoglycosides are approved by the US Food and Drug Administration (FDA) for clinical use in the United States. Of these, gentamicin, tobramycin, and amikacin are the most common parental agents. Gentamicin is often preferred because of its low cost and reliable bactericidal activity. It is administered systemically in neonatal intensive care units (NICU), mostly for prophylaxis in preterm infants, and discontinued once sepsis is ruled out <72 h. If sepsis is confirmed, treatment may continue for another 10–15 days. Tobramycin is primarily used for treating *Pseudomonas aeruginosa*-induced respiratory infections in patients with cystic fibrosis. Amikacin is particularly effective against bacteria that are resistant to other aminoglycosides, since its chemical structure makes it less susceptible to inactivating enzymes. Gentamicin and tobramycin are considered more vestibulotoxic, while amikacin, neomycin and kanamycin are considered more cochleotoxic, though each drug affects both sensory systems to varying degrees.

Almost all cells take up aminoglycosides, and most cells are able to clear these drugs from their cytoplasm relatively quickly, by mechanisms as yet undetermined, except for inner ear hair cells and renal proximal tubule cells which retain these drugs for extended periods of time (Dai et al., 2006). It is thought that this retention of aminoglycosides, plus the higher metabolic rate of hair cells and proximal tubules cells, contributes to their susceptibility to these drugs. This review will focus on the trafficking and cellular uptake of systemically-administered aminoglycosides, and their subsequent intracellular cytotoxic mechanisms. We also review factors that potentiate ototoxicity, and approaches to ameliorate aminoglycoside-induced ototoxicity.

## Functional Anatomy of the Cochlea and Kidney

### Cochlea

**Figure 2:**
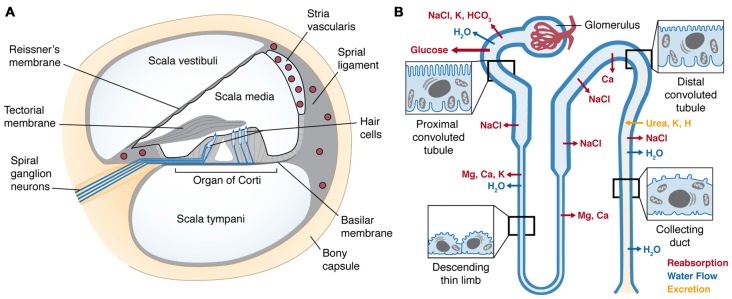
**(A)** Cross-section of the cochlear duct, illustrating the perilymph-filled scala vestibuli and scala tympani, separated from the scala media by tight junctions between adjacent cells (black line) of Reissner’s membrane and reticular lamina of the organ of Corti resting on the basilar membrane. Within the organ of Corti are four longitudinal rows of sensory hair cells (in sky blue), under the tectorial membrane. The hair cells are innervated by afferent and efferent fibers (blue lines). Within the lateral wall of the cochlea is the highly-vascularized stria vascularis (upper right); enclosing several capillary beds (red circles) lined by tight-junction-coupled endothelial cells (black lines enclosing red circles) that form the cochlear BLB. **(B)** A nephron (kidney tubule) showing the glomerulus encapsulating a single capillary bed that gathers the ultrafiltrate from blood. The proximal tubule has a brush border of microvilli that recovers the majority of essential nutrients and ions, and the distal tubule recaptures the remaining nutrients, and excretes specific ions. Sites of major ion movements are shown. Both schematic diagrams are not to relative scale.

Within the temporal bone, the cochlea is a coiled, bony tube divided into three fluid-filled compartments by two tight junction-coupled cellular barriers located on Reissner’s membrane and the basilar membrane (Figure 2A). The organ of Corti, residing on the basilar membrane, consists of sensory hair cells and adjacent supporting cells coupled together by apical tight junctions to form a reticular lamina. There are typically three rows of outer hair cells (OHCs), and a single row of inner hair cells (IHCs). The upper and lower fluid compartments, the scala vestibuli and scala tympani, respectively, are filled with perilymph similar to cerebrospinal fluid. These two compartments sandwich the inner compartment, the scala media, filled with endolymph. Uniquely, endolymph has high K^+^ concentrations due to active trafficking via Na^+^-K^+^-ATPases, Na^+^-K^+^-Cl^−^ co-transporters and rectifying potassium channels (K_ir_4.1) within the stria vascularis that generates an endocochlear potential (EP) as high as +100 mV. The stria vascularis is also a tight junction-coupled compartment and with the reticular lamina and Reissner’s membrane encloses the scala media, ensuring electrochemical separation of endolymph and perilymph (Figure 2A).

Sound pressure waves entering the cochlea tonotopically vibrate the basilar membrane, deflecting the stereocilia projecting from the apices of hair cells into endolymph. These deflections gate the mechano-electrical transduction (MET) channels on the stereociliary membrane, enabling depolarizing transduction currents that trigger the release of the neurotransmitter glutamate, which in turn induces action potentials in the innervating afferent auditory neurons (Nordang et al., 2000; Oestreicher et al., 2002). Loss of the EP reduces cochlear sensitivity to sound.

### Kidney Tubules (Nephron)

Drugs and toxins in the blood are excreted via ultra-filtration by the kidney. Renal arterial blood undergoes extravasation in kidney glomeruli, and the ultrafiltrate passes into the lumen of the proximal convoluted tubule (Figure 2B). Epithelial cells lining the proximal convoluted tubule are characterized by their extensive brush border of microvilli, maximizing the surface area available to incorporate ion channels, active transporters or exchangers and electrogenic symporters. The majority of essential nutrients, including 90% of glucose and amino acids, are resorbed from the ultrafiltrate in the proximal tubule. The tubule then descends into the medulla of the kidney and sharply reverses direction to ascend back to the kidney cortex, and is collectively called the loop of Henle. In the descending limb, water is readily resorbed, increasing the osmolarity of the ultrafiltrate, which enables additional essential ions (Na^+^, K^+^ and Cl^−^) to be resorbed in the ascending limb. As the tubule progresses into distal convoluted tubule, further cation reclamation (K^+^, Ca^2+^) occurs as H^+^ is secreted into the remaining fluid, now recognized as urine that drains into the collecting duct and bladder prior to being voided.

### Similarities and Differences between Cochlea and Kidney

There are many physiological similarities between the cochlea and kidney, principally the active transport of electrolytes or nutrients, and consequently, water follows to maintain iso-osmolarity. Gene expression analysis has identified at least 36 genes that are significantly expressed in both cochlea and kidney (Liu et al., 2004). More striking is the correlation of genetic syndromes that affect both cochlear and renal function (Izzedine et al., 2004). Both renal tubules and the stria vascularis are closely associated with basement membranes (of similar collagenous composition) that enclose blood vessels. Mutations in genes for collagen result in Alport’s syndrome characterized by progressive glomerular kidney disease and high frequency hearing loss (Gratton et al., 2005). Bartter’s syndrome results from a mutation in the gene for the protein barttin, a required subunit of voltage-gated chloride channels essential for salt and ion homeostasis in both the stria vascularis and renal ascending limb of Henle and distal tubule (Kramer et al., 2008). Hearing loss is associated in patients with lower estimated glomerular filtration rate and late chronic kidney disease (Seo et al., 2015).

Aminoglycosides are readily taken up by renal proximal tubule cells and cochlear cells (Dai et al., 2006), and more pertinently, they preferentially induce cytotoxicity in inner ear sensory hair cells and proximal tubule cells *in vivo* than for most other cell types (Humes, 1999). Other ototoxic compounds, like cisplatin and loop diuretics are also directly toxic to both organs (Humes, 1999). In addition, there is increased expression of Mpv17, a peroxisomal protein that metabolizes reactive oxygen species in renal glomeruli and the stria vascularis of the cochlea following aminoglycoside exposure (Meyer zum Gottesberge et al., 2002).

## Trafficking of Aminoglycosides *In Vivo*

### Intra-Cochlear Trafficking after Systemic Administration

In the 1980s, aminoglycosides were readily detected only in perilymph, but not endolymph, following intravenous infusion (Tran Ba Huy et al., 1986). Parental injection of gentamicin attenuated efferent inhibition of auditory neurons within 1–2 h, presumptively by blocking cholinergic activity at efferent synapses at the base of OHCs immersed in perilymph (Avan et al., 1996; Blanchet et al., 2000). The degree of the loss of inhibition may be predictive of subsequent permanent sensorineural hearing loss (Halsey et al., 2005).

*In vitro*, aminoglycosides are effective blockers of the MET channel on hair cell stereociliary membranes (Kroese et al., 1989) that, *in vivo*, are immersed in endolymph. Similar experiments then demonstrated that aminoglycosides rapidly permeate through MET channels into hair cells (Marcotti et al., 2005). Endolymph has a +80 mV potential, and when coupled with the cochlear hair cell receptor potential of −45 mV (IHCs) to −70 mV (OHCs), the potential across the apical membrane of hair cells of ~125–150 mV (Pickles, 2012). Surprisingly, adjacent supporting cells can have resting potentials between −80 mV and −100 mV (Russell and Sellick, 1978, 1983). This potent electrophoretic force likely drives cations, including aminoglycosides, across membranes through open (non-selective) cation channels with the requisite physicochemical properties for aminoglycoside permeation.

To test whether aminoglycosides could enter hair cells from endolymph *in vivo*, perfusion of the scala tympani with artificial perilymph (to prevent aminoglycoside access to the basolateral membranes of hair cells) did not visibly affect hair cell uptake of intravenously-administered aminoglycosides. However, when aminoglycoside-laden artificial perilymph was perfused though the scala tympani, hair cell uptake of aminoglycosides over their basolateral membranes was markedly reduced compared to systemic delivery (Li and Steyger, 2011). These data strongly suggest that systemic aminoglycosides are predominantly and rapidly trafficked across the blood-labyrinth barrier into the stria vascularis, and cleared into endolymph prior to entering hair cells across their apical membranes. Aminoglycosides are taken up by most other cochlear cells, including fibrocytes in the lateral wall, spiral ganglion neurons, supporting cells in the organ of Corti (Imamura and Adams, 2003; Kitahara et al., 2005; Dai et al., 2006). Aminoglycosides are cleared from non-sensory cells, but can be retained by surviving hair cells for as long as 6 months (Imamura and Adams, 2003).

### Cellular Changes Following Aminoglycoside Administration

After parental injection, basal OHCs preferentially take up aminoglycosides prior to hair cell death (Hiel et al., 1993). Multiple dosing with aminoglycosides can induce cell-specific changes in ion channel expression (see below) that may enhance drug uptake following subsequent aminoglycoside dosing, e.g., spiral ganglion cells (Kitahara et al., 2005). Aminoglycoside-induced hair cell death typically occurs in basal OHCs, and extends to IHCs and more apical OHCs with increasing cumulative dose (Forge and Schacht, 2000). The apices of dying hair cells are extruded as the surrounding supporting cell apices expand to seal the reticular lamina and prevent mixing of endolymph and perilymph, and retain optimal cochlear function in surviving hair cells. The expanded supporting cell apices, or scar, is characterized by the deposition of new junctional and cytoskeletal proteins at the site of the missing hair cell (Leonova and Raphael, 1997; Steyger et al., 1997). The hair cell bodies are typically phagocytosed by adjacent supporting cells and resident macrophages (Monzack et al., 2015).

Chronic kanamycin treatment leads to the selective loss of basal OHCs, presumptively isolating IHCs and their innervating afferent neurons which display a loss of auditory frequency selectivity and sensitivity (Dallos and Harris, 1978); however these basal IHCs also have damaged cytoskeletal networks (Hackney et al., 1990). Interestingly, significant elevations in auditory threshold occur in cochlear regions where OHCs appear morphologically intact following chronic aminoglycoside administration (Nicol et al., 1992; Koo et al., 2015). This may be due to cochlear synaptopathy, where aminoglycosides have disrupted the synapses between IHCs and their afferent neurons, as well as decreased neuronal density in the spiral ganglion of the cochlea (Oishi et al., 2015). Thus, cochlear synaptopathy may account for the greater degree of cochlear dysfunction relative to actual hair cell loss. Aminoglycosides can also induce vestibular synaptopathy, as described elsewhere in this Research Topic (Sultemeier and Hoffman, under review).

### Nephrotoxicity

In the kidney, systemic administration of aminoglycosides can induce severe toxicity in the proximal tubule that preferentially takes up aminoglycosides compared to more distal tubular regions (Dai et al., 2006). Distal tubule cells are also functionally disrupted by aminoglycoside block of magnesium and other cation channels, leading to magnesium wasting and block of ion channel function (Kang et al., 2000). Overall, disruption of kidney function tends to be short-lived, as damaged and dying proximal tubule cells are replaced through cellular proliferation (Xie et al., 2001).

## Cellular Uptake of Aminoglycosides

A major factor in susceptibility to aminoglycoside-induced toxicity is the cellular uptake of these drugs prior to inducing cell death.

### Endocytosis

Aminoglycosides are endocytosed at the apical membranes of hair cells, i.e., from endolymph, and transported to lysosomes (Hashino et al., 1997; Hailey et al., 2017). Sufficient lysosomal sequestration of aminoglycosides was hypothesized to induce lysosomal lysis, releasing both aminoglycosides and catabolic hydrolases, to initiate cell death (Hashino et al., 1997; Kroemer and Jäättelä, 2005). However, blockade of endocytosis only marginally reduced hair cell uptake of aminoglycosides and did not prevent hair cell death (Alharazneh et al., 2011; Hailey et al., 2017). Aminoglycosides in the cytoplasm can be sequestered by endosomes prior to being trafficked to lysosomes, a novel form of autophagy (Hailey et al., 2017). Impeding the lysosomal trafficking of aminoglycoside-laden endosomes potentiated drug-induced hair cell death, suggesting that endosomal sequestration of aminoglycosides can partially protect hair cells (Hailey et al., 2017).

In the kidney, megalin, also known as the low density lipoprotein-related protein 2 (LRP2), associates with cubulin, a co-receptor, and when bound to aminoglycosides, the complex is endocytosed (Christensen and Nielsen, 2007). Megalin-deficient mice are profoundly deaf by 3 months of age (early-onset presbycusis) and have reduced renal uptake of aminoglycosides (Schmitz et al., 2002; Köonig et al., 2008). In the cochlea, megalin is expressed near the apical (endolymphatic) membrane of strial marginal cells, but is not expressed in cochlear hair cells (Köonig et al., 2008). This suggests that megalin-dependent endocytosis of aminoglycosides by marginal cells, i.e., clearance from endolymph, could provide partial otoprotection for hair cells.

### Ion Channels

Aminoglycosides can permeate many ubiquitously-expressed non-selective cation channels with the requisite physicochemical properties to accommodate aminoglycosides. In addition to the inner ear and kidney, aminoglycosides are readily taken up by sensory neurons in the dorsal root and trigeminal ganglia, linguinal taste receptors, and sensory neurons of hair follicles (Dai et al., 2006). Each location expresses a variety of aminoglycoside-permeant ion channels, including non-selective Transient Receptor Potential (TRP) cation channels.

In the inner ear, aminoglycosides readily permeate the non-selective MET cation channel expressed on the stereociliary membranes of hair cells (Marcotti et al., 2005). Although the identity of MET channels (pore diameter ~1.25 nm) remain uncertain, their electrophysiological properties are well-characterized and major components, including transmembrane channel-like (TMC) 1 and TMC2 proteins, have been identified (Farris et al., 2006; Kawashima et al., 2011). Mutations in myosin VIIA, another component of the MET complex, dysregulate MET channel conductance, reducing drug uptake by hair cells (Kros et al., 2002). Extracellular cadherin-23 and protococadherin-15 proteins form the stereociliary tip-links that mechanically gate the MET channel, and mutation in these genes reduced aminoglycoside uptake, prolonging hair cell survival compared to wild-type hair cells (Vu et al., 2013). The conductance of MET channels is modulated by extracellular [Ca^2+^], and reduced by channel blockers like amiloride, curare or benzamil; each can reduce hair cell uptake of aminoglycosides and/or prolong hair cell survival (Marcotti et al., 2005; Coffin et al., 2009; Alharazneh et al., 2011; Hailey et al., 2017). Increasing the membrane potential difference between the extracellular fluid and the negatively-polarized cytoplasm increases cellular uptake of the cationic aminoglycosides in hair cells and renal cells (Marcotti et al., 2005; Myrdal and Steyger, 2005).

Several identified non-selective cation channels are candidates for aminoglycoside permeation, particularly TRP channels with pore diameters sufficient to admit the maximal cross-sectional diameter of aminoglycosides (0.8–0.9 nm). The TRP vanilloid receptor 1, TRPV1, was identified using a number of channel modulators (Myrdal and Steyger, 2005). TRPV1 is activated by heat (<43°C), and is also stimulated by capsaicin (or analogs) and protons (Caterina et al., 1997; Vellani et al., 2001). TRPV1 has a pore diameter of ~1 nm (Jara-Oseguera et al., 2008) that can be further dilated by agonists (Bautista and Julius, 2008). Capsaicin activation of cells heterologously expressing TRPV1 induces rapid cell death in streptomycin-containing culture media (Caterina et al., 1997), suggestive of aminoglycoside permeation and subsequent cytotoxicity. TRPV1 is expressed by hair cells and plays a critical role in cisplatin-induced toxicity (Zheng et al., 2003; Mukherjea et al., 2011).

TRPV4 channels are temperature-sensitive (25–34°C) cation channels that are also activated by osmotic swelling of cells, and chemically activated by 4α-phorbol 12,13-didecanoate (Liedtke et al., 2000; Vriens et al., 2004). TRPV4 has a large pore diameter (Shigematsu et al., 2010), is expressed on the apical surface of hair cells, and is aminoglycoside-permeant when overexpressed in kidney proximal tubule cell lines (Karasawa et al., 2008). Low [Ca^2+^] increase the open probability of TRPV4 channels (Banke, 2011). Crucially, endolymph has low [Ca^2+^] (Wangemann and Schacht, 1996), increasing the likelihood of aminoglycosides entering the cytoplasm of cells with membranous TRPV4 channels bathed by extracellular endolymph.

TRPA1 (TRP channel, subfamily A, member 1) channels are inflammatory, irritant and oxidative stress sensors (Kwan et al., 2006; Macpherson et al., 2007; Bessac et al., 2008), and appear to reside in the basolateral membrane of OHCs (Stepanyan et al., 2011). TRPA1 channels have a pore diameter of 1.1 nm and show agonist-induced dilation (to ~1.4 nm; Karashima et al., 2010), larger than the molecular diameter of aminoglycosides. The TRPA1 agonists, cinnamaldehyde and 4-hydroxynonenal (4-HNE), both increased OHC uptake of aminoglycosides, presumptively across their basolateral membranes *in vitro* (Stepanyan et al., 2011), suggesting that endogenous intracellular activation of basolateral TRPA1 channels due to oxidative stress, induced by noise (Henderson et al., 2006) or aminoglycoside exposure (Lesniak et al., 2005), could augment hair cell uptake of aminoglycosides from the scala tympani. The promiscuous permeation of several non-selective cation channels by aminoglycosides suggest that additional aminoglycoside-permeant channels will be identified (based on permeation by other cationic organic compounds). These include connexins (or gap junctions), pannexins (hemi-channels), canonical TRPC3 with a large inner chamber (~6 nm diameter) and P2X channels among others (Weber et al., 2004; Mio et al., 2007; Crumling et al., 2009; Torres et al., 2017).

### Transporters

An electrogenic Na^+^-ligand symporter, sodium glucose transporter 2 (SGLT2), resorbs 90% of lumenal glucose from renal ultra-filtrate in proximal tubules (Kanai et al., 1994). Inhibitors of SGLT2 inhibitors significantly block renal glucose reabsorption (Ghosh et al., 2012). SGLT2 has a large, hydrophilic and elastic vestibule, with an internal pore diameter of 3 nm, and an exit pore (into cytosol) of 1.5–2.5 nm, sufficient for aminoglycoside permeation. Aminoglycosides are complex sugars connected by glycosidic linkage (Neu and Gootz, 1996), and overexpression of SGLT2 in cell lines increased cellular uptake of aminoglycosides and exacerbated subsequent cytotoxicity (Jiang et al., 2014). Inhibition of SGLT2 by phlorizin reduced aminoglycoside-induced toxicity in proximal tubule cells *in vitro* and *in vivo*. However, phlorizin inhibition of SGLT2 *in vivo* did not reduce cochlear loading of aminoglycosides, potentially due to low cochlear expression levels of SGLT2, and/or by the phlorizin-induced elevation of serum aminoglycoside levels (Jiang et al., 2014). Since acute pharmacological inhibition or genomic loss of SGLT2 function did not affect auditory function (Jiang et al., 2014), this suggests that aminoglycoside (and glucose) trafficking across the blood-labyrinth barrier is accomplished by other molecular mechanisms, such as the facilitated glucose transporters (GLUTs; Ando et al., 2008). It is not yet known whether GLUTs are aminoglycoside-permeant and their pore dimensions remain to be determined, although it is known that the stria vascularis and organ of Corti both express GLUT5 (Belyantseva et al., 2000).

## Noise and Aminoglycosides

Loud sounds affect almost all cochlear cell types, including physically disrupting hair cell stereocilia, mitochondria, and the loss of synapses between hair cells and afferent neurons leading to transient and permanent hearing losses that accelerate the onset of presbycusis (Bohne et al., 2007; Kujawa and Liberman, 2015). Exposure to loud sounds synergistically potentiates the ototoxicity of aminoglycosides (Brown et al., 1978), presumptively by the summation of reactive oxygen species generated by each insult alone (Kopke et al., 1999). Loud sounds also break tip-links between stereocilia, closing the mechanically-gated MET channels (Husbands et al., 1999; Kurian et al., 2003). Sound levels that induce temporary threshold shifts (TTS) enhanced OHC uptake of aminoglycosides in mice, yet significantly reduced the number of tip links between OHC stereocilia (Li et al., 2015). This indicates that increased uptake of aminoglycosides by hair cells occurs by a mechanism distinct from MET channels. Loss of tip links would hyperpolarize hair cells, increasing the electrophoretic driving force from endolymph into hair cells, facilitating aminoglycoside permeation of other non-selective cation channels (Li et al., 2015).

The synergistic ototoxicity of loud sounds and aminoglycosides is not confined to simultaneous exposure. Loud sound exposure weeks prior to treatment with aminoglycosides can also potentiate aminoglycoside-induced hearing loss (Ryan and Bone, 1978). Low doses of aminoglycosides prior to loud sound exposure can *reduce* hearing loss compared to those exposed to loud sounds alone, a phenomenon called preconditioning (Fernandez et al., 2010), yet this is dependent on dosing regimen, age of treatment, anti-oxidant defenses and genetic background (Lautermann and Schacht, 1996; Kopke et al., 1999; Ohlemiller et al., 2011). Identifying the physiologic or genetic mechanisms behind these variations could establish who is at elevated risk of acquired hearing loss. These studies are clinically relevant as aminoglycosides are systemically administered in NICU, where sustained levels of higher ambient sound levels (Williams et al., 2007; Garinis et al., 2017b) could increase the risk of aminoglycoside-induced cochleotoxicity.

## Co-Therapeutics that Potentiate Aminoglycoside-Induced Ototoxicity

Most prominent are the loop diuretics, administered to reduce high blood pressure and edema. In sufficient dosing it will cause temporary, or in some cases permanent, hearing loss. Loop diuretics block Na^+^-K^+^-Cl^−^ co-transporter trafficking of potassium into marginal cells, resulting in a loss of the EP (Higashiyama et al., 2003). This drug-induced loss of EP facilitates (by unknown mechanisms) greater entry of aminoglycosides into endolymph, and once the EP is restored, rapid and greater hair cell death (Rybak, 1982; Tran Ba Huy et al., 1983). This outcome is used experimentally to accelerate experimental timeframes in studies of cochlear repair and regeneration processes in mammals (Taylor et al., 2008).

Vancomycin, a glycopeptide antibiotic commonly-prescribed in the NICU (Rubin et al., 2002), can enhance aminoglycoside-induced ototoxicity in preclinical models (Brummett et al., 1990). Vancomycin alone induced acute nephrotoxicity in ~1–9% of neonates (Lestner et al., 2016), yet conflicting evidence for stand-alone vancomycin-induced ototoxicity in humans and preclinical models suggest that potential confounders and clinical settings (e.g., inflammation, see “Inflammation and Aminoglycosides” Section below) need to be considered in the analyses.

## Inflammation and Aminoglycosides

Until recently, the inner ear has been considered an immunologically-privileged site, as major components of the inflammatory response (e.g., immune cells, antibodies) are largely excluded by the blood-labyrinth barrier from inner ear tissues (Oh et al., 2012). This barrier is considered to reside at the endothelial cells of the non-fenestrated blood vessels traversing through the inner ear. However, recent pioneering studies show active inner ear participation in classical local and systemic inflammatory mechanisms, with unexpected and unintended consequences.

Middle ear infections increase the permeability of the round window to macromolecules, enabling pro-inflammatory signals and bacterial endotoxins in the middle ear to penetrate the round window into cochlear perilymph (Kawauchi et al., 1989; Ikeda et al., 1990). Spiral ligament fibrocytes lining the scala tympani respond to these immunogenic signals by releasing inflammatory chemokines that attract immune cells to migrate across the blood-labyrinth barrier into the cochlea, especially after hair cell death—another immunogenic signal (Oh et al., 2012; Kaur et al., 2015), and reviewed elsewhere in this Research Topic (Wood and Zuo, 2017). In addition, perivascular macrophages adjacent to cochlear blood vessels (Zhang et al., 2012), and supporting cells in the organ of Corti, exhibit glial-like (anti-inflammatory) phagocytosis of cellular debris following the death of nearby cells (Monzack et al., 2015). These data imply that inner ear tissues can mount a sterile inflammatory response similar to that observed after noise-induced cochlear cell death (Hirose et al., 2005; Fujioka et al., 2014).

In contrast, systemic inflammatory challenges experimentally do not generally modulate auditory function (Hirose et al., 2014b; Koo et al., 2015), with meningitis being a major exception. Nonetheless, systemic inflammation changes cochlear physiology, vasodilating cochlear blood vessels, although the tight junctions between endothelial cells of cochlear capillaries appear to be intact (Koo et al., 2015). Systemic inflammation also induces a 2–3 fold increase in the permeability of the blood-perilymph barrier (Hirose et al., 2014a), and increased cochlear levels of inflammatory markers (Koo et al., 2015). Systemic administration of immunogenic stimuli together with aminoglycosides triggered cochlear recruitment of mononuclear phagocytes into the spiral ligament over several days (Hirose et al., 2014b). Thus, cochlear tissues participate in the systemic inflammatory response induced by systemic immunogenic stimuli, as well as middle ear or intra-cochlear immunogenic stimuli from bacteria or cellular debris.

To date, most studies of aminoglycoside-induced ototoxicity have been conducted in healthy preclinical models, unlike the administration of aminoglycosides to those with severe infections (and consequent inflammation) in clinical settings. Preclinical models with systemic inflammation, induced by low doses of bacterial lipopolysaccharides displayed increased cochlear uptake of aminoglycosides, and enhanced levels of cochleotoxicity without altered serum drug levels (Koo et al., 2015). Inflammation also potentiates cisplatin-induced ototoxicity (Oh et al., 2011). The potential mechanisms by which systemic inflammation enhances aminoglycoside-induced ototoxicity is discussed elsewhere in this Research Topic (Jiang et al., under review). Much further work is required to unravel how inflammation affects: (i) cochlear physiology; and (ii) repair of cochlear lesions following noise exposure or ototoxicity, as discussed elsewhere in this Research Topic (Kalinec et al., 2017).

## Intracellular Mechanisms of Aminoglycoside Cochleotoxicity

Although molecular mechanisms involving reactive oxygen species, c-Jun N-terminal kinase (JNK) and caspase signaling cascades have been described elsewhere in detail (Ylikoski et al., 2002; Matsui et al., 2004; Lesniak et al., 2005; Coffin et al., 2013), there are still gaps in understanding how aminoglycosides induce cytotoxicity. Below, we focus how mitochondria and endoplasmic reticula (ER) are also primary induction sites for aminoglycoside-induced cytotoxicity.

As antimicrobial agents, aminoglycosides target bacterial ribosomes and induce misreading during protein synthesis (Cox et al., 1964; Davies and Davis, 1968). A genetic study demonstrated that aminoglycoside susceptibility can be transmitted by matrilineal descent, suggesting mitochondrial inheritance (Hu et al., 1991). Analysis of mitochondrial ribosomes revealed that the A1555G polymorphism in 12S rRNA is associated with aminoglycoside-induced hearing loss (Prezant et al., 1993). Other mitochondrial 12S rRNA mutations, including C1494T and T1095C, also increase susceptibility to aminoglycoside ototoxicity (Zhao H. et al., 2004; Zhao L. et al., 2004). Mitochondrial mutations that lead to 12S rRNA binding with a higher affinity to aminoglycosides can cause misreading of the genetic code and mistranslated proteins is a primary mechanism of cytotoxicity (Hobbie et al., 2008; Qian and Guan, 2009). The variety of novel aminoglycoside-interacting proteins involved in mitochondrial respiration, in addition to other ribosomal or nuclear-targeting proteins with a basic-peptide motif, supports the hypothesis that mitochondrial function is a primary site of aminoglycoside-induced cytotoxicity (Kommareddi and Schacht, 2008). Additionally, mutations in *TRMU*, a nuclear modifier gene, can modulate the phenotypic manifestation of deafness-associated 12S rRNA mutations (Guan et al., 2006).

Aminoglycosides also induce ribotoxic stress by binding to cytosolic rRNA to inhibit protein synthesis in eukaryotes (Francis et al., 2013). Aminoglycosides have a higher binding affinity (K_d_ of 1.7 μM) for the 28S rRNA than for 12S rRNA, a concentration readily reached in hair cells at clinically-relevant concentrations (Marcotti et al., 2005; Francis et al., 2013). Through these mechanisms, aminoglycosides could further inhibit eukaryotic protein synthesis, and activate stress-induced apoptosis mechanisms.

Many cytosolic proteins also bind to aminoglycosides (Karasawa et al., 2010). Calreticulin, an ER chaperone protein (Horibe et al., 2004; Karasawa et al., 2011), assists in protein folding, quality control and degradation (Williams, 2006). Although calreticulin is ubiquitously expressed, it is highly expressed in cochlear marginal cells, and hair cell stereocilia (Karasawa et al., 2011). Calreticulin binds to Ca^2+^ and aminoglycosides at the same site (Karasawa et al., 2011). Aminoglycoside binding to calreticulin likely disrupts the chaperone activity, homeostatic calcium buffering or regulation of calreticulin activity in these cells that becomes cytotoxic (Bastianutto et al., 1995; Mesaeli et al., 1999). Aminoglycosides also dysregulate intracellular Ca^2+^ stores to facilitate toxic transfers of Ca^2+^ from the ER into mitochondria via inositol-1,4,5-triphosphate (IP_3_) receptors (Esterberg et al., 2013). This, in turn, elevates mitochondrial Ca^2+^ that underlies elevated levels of both mitochondrial oxidation and cytoplasmic ROS prior to cell death (Esterberg et al., 2016).

Aminoglycosides can bind to another ER protein, CLIMP-63 (Karasawa et al., 2010), thought to anchor microtubules to the ER (Sandoz and van der Goot, 2015). CLIMP-63 is highly expressed in cultured HEI-OC1 cells derived from the murine organ of Corti. Aminoglycosides oligomerize CLIMP-63 that then bind to 14-3-3 proteins; knockdown of either CLIMP-63 or 14-3-3β suppressed aminoglycoside-induced apoptosis (Karasawa et al., 2010). 14-3-3 proteins are implicated in both pro- and anti-apoptosis mechanisms that involve p53, tumor suppressor gene, and binding of 14-3-3 proteins to MDMX, a negative regulator of p53, induces apoptosis (Okamoto et al., 2005). Thus, aminoglycoside binding to CLIMP-63 may promote p53-dependent apoptosis via 14-3-3 inhibition of MDMX.

## Potential Clinical Approaches to Reduce Aminoglycoside Uptake or Ototoxicity

Over 5% of the world’s population, ~360 million people, have hearing loss (WHO, 2012; Blackwell et al., 2014). Two major otoprotective strategies against aminoglycoside-induced hearing loss have been proposed. One is to reduce drug uptake by cells to prevent cytotoxicity; another is to interfere with mechanisms of aminoglycoside-induced cytotoxicity.

### Reducing Cellular Uptake of Aminoglycosides

In the NICU, aminoglycosides, especially gentamicin, are often obligatory treatments to treat life-threatening sepsis (Cross et al., 2015). NICU environments have loud ambient sound levels (Williams et al., 2007; Garinis et al., 2017b), and a significantly increased incidence of hearing loss in NICU graduates (Yoon et al., 2003) that may be due to the synergistic effect of ambient sound levels increasing cochlear uptake of aminoglycosides (Li et al., 2015). Thus, efforts to reduce ambient sound levels in the NICU will be welcomed.

Inflammation caused by severe bacterial infections also increase cochlear uptake of aminoglycosides and subsequent ototoxicity (Koo et al., 2015). Administration of anti-inflammatory agents prior to or during aminoglycoside treatment may be effective as for etanercept, an antibody, that blocks the pro-inflammatory signaling receptor TNFα, in ameliorating noise-induced hearing loss (Arpornchayanon et al., 2013). Etanercept and perhaps other anti-inflammatory agents can reduce cochlear inflammation (Satoh et al., 2002), and could also reduce cochlear uptake of aminoglycosides, to better preserve auditory function, similar to glucocorticoids restoring auditory function by improving the ion homeostatic (mineralocorticoid) activity of the blood-labyrinth barrier (MacArthur et al., 2015).

The zebrafish lateral line is an excellent model to conduct high throughput screening of compounds that protect hair cells from ototoxicity (Harris et al., 2003). A recent screening of over 500 natural compounds identified four novel bisbenzylisoquinoline derivatives, berbamine, E6 berbamine, hernandezine, and isotetrandrine, as otoprotective agents that reduce hair cell uptake of aminoglycosides (Kruger et al., 2016; Kirkwood et al., 2017). Since these compounds block the aminoglycoside-permeant MET channels, these drugs are also expected be effective in reducing mammalian hair cell uptake of aminoglycosides *in vitro*, yet, verification is crucial (Majumder et al., 2017). It is also crucial to test *in vivo* following local or systemic administration to ensure these compounds can enter the compartmentalized endolymphatic fluids.

### Reducing Aminoglycoside Cytotoxicity

Several anti-oxidants like *N*-acetylcysteine, D-methionine and edaravone reduce aminoglycoside-induced ototoxicity in preclinical models (Somdaş et al., 2015; Campbell et al., 2016; Turan et al., 2017), suggesting that drug-induced generation of reactive oxygen species leads to aminoglycoside-induced ototoxicity. Several anti-oxidants show otoprotection against both aminoglycosides and cisplatin, implying that induction of oxidative stress is a shared mechanism of cytotoxicity for these ototoxins (Lorito et al., 2011; Tate et al., 2017). If this is the case, then dosing regimens reducing cisplatin-induced ototoxicity may also translate to being otoprotective for aminoglycoside-induced ototoxicity. An *in vitro* screen to test for the otoprotective (or ototoxic) properties of antioxidants in the organ of Corti explants is described elsewhere in this Research Topic (Noack et al., 2017).

Another innovative strategy is to develop aminoglycosides like apramycin with minimal affinity for eukaryotic mitochondrial ribosomes while retaining strong activity against clinical pathogens (Matt et al., 2012). An alternative, pioneering method is to modify specific amine groups of sisomicin (a biosynthetic precursor of gentamicin), generating several designer aminoglycosides. One modified aminoglycoside, N1MS, displayed significantly reduced ototoxicity while retaining bactericidal efficacy in preclinical models (Huth et al., 2015).

Acetylation of histones, proteins required for chromatin regulation of gene transcription, is associated with gene transcription activation, and histone deacetylases (HDACs) regulate this process. Aminoglycosides also hypo-acetylate histones, reducing transcription factor binding to DNA, causing decreased levels of gene expression (Chen et al., 2009). Since HDACs remove histone acetylation, inhibitors of HDACs were found to provide otoprotection in cochlear explants (Chen et al., 2009), but not *in vivo* (Yang et al., 2017). In contrast, systemic HDAC inhibition using suberoylanilide hydroxamic acid (SAHA) resulted in almost complete protection against *combined* kanamycin and furosemide-induced ototoxicity, and this mechanism involved activating the NF-κB pathway (Layman et al., 2015), indicating that verification of candidate otoprotective agents requires testing in models that more closely resemble clinical situations, i.e., chronic dosing with aminoglycosides, preferably in the setting of inflammation (Koo et al., 2015). In the same vein, interfering with cell death signaling pathways also promoted acute hair cell survival and attenuated drug-induced hearing loss following chronic aminoglycoside dosing (Ylikoski et al., 2002).

Another promising approach involves activating heat shock proteins (HSPs), including HSP70, to promote hair cell survival against aminoglycoside ototoxicity (Taleb et al., 2008). Heat shock induces expression and secretion of HSP70 by supporting cells to effect otoprotection of hair cells (May et al., 2013). Intriguingly, exposure to sound sufficient to transiently stress the cochlea (without inducing permanent hearing loss, i.e., preconditioning) upregulated the expression of HSP70 (and HSP32) expression to significantly reduce aminoglycoside-induced hearing loss in preclinical models (Roy et al., 2013). Further discussion of the pro-survival and cell death factors influencing hair cell survival and hair cell death via autonomous and non-autonomous mechanisms are discussed elsewhere in this Research Topic (Francis and Cunningham, 2017).

## Conclusion

Aminoglycoside antibiotics remain crucial pharmacotherapeutics for severe bacterial infections, despite their known side effects and the emergence of other (more labile) classes of broad-spectrum antibiotics. Aminoglycosides are also preferred due to their robust stability at ambient temperatures when used by itinerant healthcare providers in the field, and because of their bactericidal efficacy against bacteria resistant to other antibiotics. Increasing our understanding of aminoglycoside-induced (oto)toxicity requires greater insight into the mechanisms of cellular uptake kinetics, transcellular trafficking and intracellular disruption of physiological activities by aminoglycosides, especially in models that better mimic clinical settings such as exposure to higher levels of ambient sounds, co-therapeutics and/or inflammation that potentiate the degree of ototoxicity. Modifying dosing protocols, the structure of current aminoglycosides, and/or increased verification of candidate otoprotective agents could all enable aminoglycosides to be used more readily with reduced risks of lifelong ototoxicity in hospital.

## Author Contributions

This review was conceived, written and edited by each of the authors (MJ, TK and PSS).

## Conflict of Interest Statement

The authors declare that the research was conducted in the absence of any commercial or financial relationships that could be construed as a potential conflict of interest.
